# Development and validation of molecular markers for characterization of *Boehmeria nivea *var. *nivea *and *Boehmeria nivea *var. *tenacissima*

**DOI:** 10.1186/1749-8546-5-40

**Published:** 2010-11-29

**Authors:** Chuan-I Li, Shu-Jiau Chiou, Teng-Soung Tong, Cheng-Yu Lee, Lain-Tze Lee, Ching-Ming Cheng

**Affiliations:** 1 Biomedical Technology and Device Research Laboratories, Industrial Technology Research Institute, Hsinchu 300, Taiwan; 2 Graduate Institute of Chinese Pharmaceutical Science, China Medical University, Taichung 404, Taiwan; 3 Department of Life Sciences, Tzu-Chi University, Hualien 970, Taiwan

## Abstract

**Background:**

The root of *Boehmeria *spp (ramie) is a hepatoprotective Chinese herbal medicine. Medicinal properties vary between *Boehmeria nivea *var. *nivea *and *Boehmeria nivea *var. *tenacissima*, which are local species found in Taiwan. As commercial preparations may use either species, there is a need for a rapid and simple assay to identify variants for quality control.

**Methods:**

Four methods were developed and tested for their applicability in differentiating the two species. These methods were random amplified polymorphic DNA (RAPD); sequence characterized amplified regions (SCAR); single nucleotide polymorphisms (SNP) and cleaved amplified polymorphic sequences (CAPS).

**Results:**

Three RAPD markers were developed that produced unique bands in *B. nivea *var. *tenacissima *and *B. nivea *var. *nivea*. Based on sequenced RAPD bands, one SCAR marker was developed that produced a single DNA band in *B. nivea *var. *nivea*. Two SNP markers differentiated between *B. nivea *var. *nivea *and *B. nivea *var. *tenacissima *based on single nucleotide substitutions. A pair of CAPS oligonucleotides was developed by amplifying a 0.55-kb DNA fragment that exhibited species-specific digestion patterns with restriction enzymes *Alf *III and *Nde *I. Consistent results were obtained with all the four markers on all tested *Boehmeria *lines.

**Conclusion:**

The present study demonstrates the use of the RAPD, SCAR, SNP and CAPS markers for rapid identification of two closely related *Boehmeria *species.

## Background

The root of *Boehmeria *species (Urticaceae), namely *Boehmeria nivea *var. *nivea *is a hepatoprotective Chinese herbal medicine [1] as well as an antioxidant and anti-inflammatory agent [2]. Sancheti and colleagues have reported its glycosidase and cholinesterase inhibition properties as an anti-diabetic herb to lower blood glucose and cholesterol levels [3]. Compared to *B. nivea *var. *nivea*, *B. nivea *var. *tenacissima *is more hepatoprotective on hepatitis B-induced liver damage [4]. As commercial preparations may consist of one or the other variants, there is a need for rapid and simple assays to identify variants for the purpose of both commercial production and quality control. Whereas today's methods rely primarily on morphological observations, molecular genetics are a more precise tool, less susceptible to user bias.

Based on four molecular approaches, namely random amplified polymorphic DNA (RAPD), sequence characterized amplified region (SCAR), single nucleotide polymorphism (SNP) and cleaved amplified polymorphic sequence (CAPS), we developed and evaluated a set of authentication techniques for the *Boehmeria *species and help conserve Chinese medicinal plants in Taiwan.

RAPD is a modified polymerase chain reaction (PCR) technique involving multiple oligonucleotide primers. The resulting amplified DNA markers are random polymorphic segments with band sizes from 100 to 3000 bp depending upon the genomic DNA and the primer. SCARs are DNA fragments amplified by using specific 15-30 bp primers, designed from nucleotide sequences established in cloned RAPD fragments. By using longer PCR primers, SCARs have a higher rate of reproducibility than RAPDs. SNP analysis is more specific still but requires sequencing to identify the different nucleotides.

CAPS polymorphisms are differences in restriction fragment lengths caused by SNPs that create or abolish restriction endonuclease recognition sites in PCR amplicons. All of these markers are locus-specific with a wide range of applicability in gene mapping and marker-assisted selection [5-7]. This article describes the main results of the study.

## Methods

### Plant materials

Eight lines of *B. nivea *var. *nivea *and *B. nivea *var. *tenacissima *were collected from various locations of Taiwan and identified by one of the authors (TST), based on the criteria that *B. nivea *var. *nivea *has a white-grey color with obvious pubescence in their ventral leaf surface and *B. nivea *var. *tenacissima *has a light green-grey color [8]. Four collections, namely CY1 (Chi-Yi-1), CY2 (Chi-Yi-2), CY3 (Chi-Yi-3) and HCn (Hsin-Chu-n) belong to *B. nivea *var. *tenacissima *and the other four, namely HCd (Hsin-Chu-d), TC (Tai-Chung), CY (Chi-Yi) and TARI (Taiwan Agricultural Research Institute) are local variants of *B. nivea *var. *nivea*.

DNA extraction was performed according to the method described by Arasl *et al. *[9]. Briefly, 100 mg fresh leaves were ground in liquid nitrogen and transferred to tubes containing 5 mL CTAB/PVPP extraction buffer which consisted of 0.1 M Tris HCl, 1 M NaCl, 20 mM EDTA, 1% hexadecyl trimethylammonium bromide (CTAB; w/vol) and 1% polyvinylpolypyrrolidone (PVPP; w/vol). The mixture was incubated at 65°C for 20 minutes and extracted with an equal volume of chloroform/isoamylalcohol (24:1). After centrifugation (8,000×*g*, Sigma 3-18 K, Germany) for 5 minutes, the supernatant was transferred to a clean tube and precipitated with two volumes of precipitation buffer (50 mM Tris HCl, 4 mM NaCl, 10 mM EDTA and 1% CATB) at 10, 000×*g *for 20 minutes. The pellet was re-suspended in 350 μL 1.2 M NaCl and incubated with 10 mg/mL RNase at 37°C for 30 minutes. After extraction with an equal volume of chloroform/isoamylalcohol (24:1), the DNA pellet was re-precipitated with ice-cold isopropanol, washed with 70% ethanol, vacuum dried and dissolved in 200 μL TE buffer.

### RAPD

RAPD reactions [10] were carried out in a final volume of 25 μL containing 1 unit Taq DNA polymerase, 100 μM dNTP mixture, 10 mM Tris HCl, 1.5 mM MgCl_2_, 1.0 μM primer and 10-20 ng template DNA. Amplification was performed in a PCR machine (Thermocycler 2100, PerkinElmer, USA) at 94°C for two minutes followed by 40 cycles of 30 seconds at 94°C, 40 seconds at 36°C, 45 seconds at 72°C and a final stage of five minutes at 72°C. The amplification products were maintained at 4°C and resolved in 1.5% agarose gel followed by ethidium bromide staining and visualization with UV light for photography. The amplified DNA fragments, RP-S343-1.1, RP-S343-0.9 and RP-S62-0.6 were used for oligonucleotide design (Table 1). To avoid sequences that would produce internal secondary structures, we checked primers with Oligo 6 software (National Sciences, USA)

**Table 1:** Primers used for marker analysis

Technique	Annealing temperature (°C)	Name of the primer	Sequence	Number of polymorphic bands	Marker length (kb)
RAPD	35-40	S62	GTGAGGCGTC	1	0.6
		S343	TCGTGCGGGT	2	0.9,1.1
SCAR	55-60	SR-S343-F1	CTCTTGAGCAATCCAAATGTTTTGTTATCA		
		SR-S343-R1	CATAAATCACTTTATAACATAACGAGCTCGTATT	1	1.03
		SR-S343-R2	CGCGACAGAGGGGTTTTCTTTCTATTA	1	0.95
		SR-S343-R3	AGACGCCTCACTTTGATAGACATGAGTTTA	1	0.89
SNP	50-60	Sn-S62-a	CGACAGTAAACATAAAAACCG	1	1*
		Sn-S62-b	CTGTTACCATTGGCTCTTTACC		
CAPS	60-67	CP-S62-f	TCGTGCGGGTCATAGTACCCCGAGACAAGAGGCCAAAA	2	0.25, 0.3 (*Afl *III)
		CP-S62-r	TGTAATACGAAAGTTTAAGTCTCTTTTCTTAGTC		0.2, 0.35 (*Drd *I)

* Only one base with different colors. The fluorescent dyes are assigned to the individual ddNTPs. Each ddNTP is labeled with a different color, ddATP green, ddCTP blue, ddGTP black, and ddTTP red.

### SCAR

Three pairs of oligonucleotides were used in the SCAR assays [11], namely forward oligonucleotide SR-S343-F1 and the three reverse oligonucleotides SR-S343-R1, SR-S343-R2 and SR-S343-R3 (Table 1). The SCAR reaction was performed with an initial denaturation step at 95°C for five minutes, followed by 35 cycles of 94°C for two minutes, 60°C for one minute, 72°C for one minute and a 10-minute final extension at 72°C.

PCR fragments were cloned with TA cloning technology using pGEM-T-Easy vectors (Promega, USA) and used to transform the *Escherichia coli *strain XL-2 Blue (Stratagene, USA). DNA sequence analysis was carried out with the BLAST sequence analysis programs at the National Center for Biotechnology Information (NCBI) [12]. Alignments were edited with the online ClustalW program from DNA Data Bank of Japan [13].

### SNP

A sequence from a RAPD DNA fragment, namely RP-S62-0.6, was chosen for SNP detection. The procedures were performed according to the manufacturer's instructions [14] with one modification, i.e. the mixture solution was diluted 1:8 with magnesium buffer (400 mM Tris pH 9 and 10 mM MgCl_2_). Each reaction contained 0.5 μL of the SNaPshot™ Multiplex Ready Reaction Mix (Applied Biosystems, USA), 2.0 μL of PCR product, 1.0 μL of extension primers and water up to 10 μL. Thermal cycling and post-extension were run on an ABI Prism 3100 Genetic Analyzer (Applied Biosystems, USA).

### CAPS

CAPS analyses were performed according to published methods [15]. The RP-S62-0.6 DNA fragment was amplified from the eight *Boehmeria *lines with primers CP-S62-f and CP-S62-r. DNA fragments were digested with restriction enzymes (i.e. *Afl *III, *Bsr *FI, *Msp *I, *Drd *I and *Nde *I) and separated on a 1.5% agarose gel for polymorphism detection.

### Quality control

A mixture of DNA was used to identify the basis of all the markers for quality control. Samples contained DNA from both *B. nivea *var. *nivea *and *B. nivea *var. *tenacissima *in the ratios of 9:1, 8:2, 7:3, 6:4, 5:5, 4:6, 3:7, 2:8 and 1:9 respectively.

## Results and Discussion

### RAPD markers for quick screening

**Figure 1:**
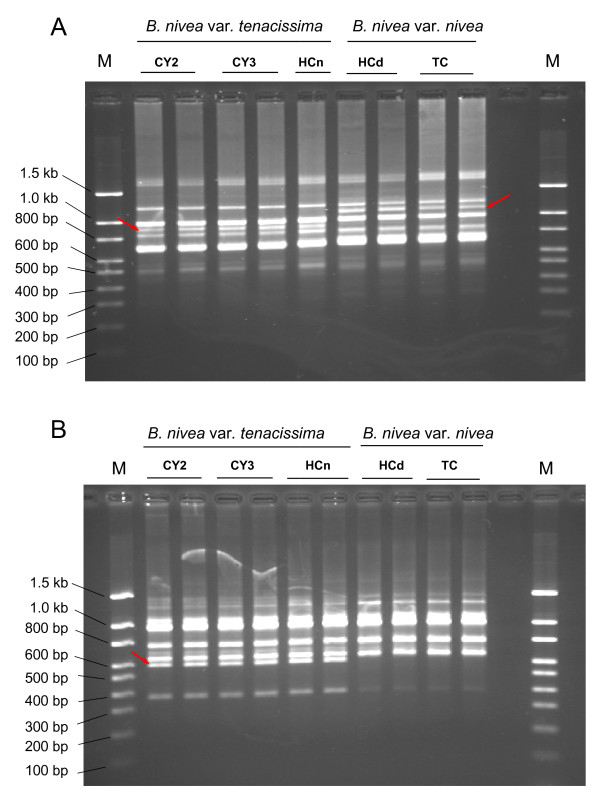
**RAPD fingerprint patterns generated with primer S343 and S62**. **A **RAPD profile generated with primer S343. The unique banding patterns are indicated by arrows. Representative samples of both species are shown on the top of lanes. The numbers on the left indicate the size of DNA standards. M: DNA markers. **B **RAPD profile generated with primer S62.

Out of a set of 100 RAPD primers, two primers, namely S343 and S62, produced clear reproducible unique patterns easily distinguishable from one another (Figure 1A and Figure 1B) and were selected for further investigation. The RAPD marker S343 produced two polymorphic bands of 1.1 kb and 0.9 kb unique to the *B. nivea *var. *nivea *and *B. nivea *var. *tenacissima *species, respectively, while the S62 RAPD marker produced one polymorphic band of 0.6 kb in *B. nivea *var. *tenacissima *but none in *B. nivea *var. *nivea*. The unique bands were amplified, cloned and sequenced. No significantly related genes were found in the GenBank database.

RAPD analysis is fast and economical [16] as long as suitable primers are available. In the present study, only two primers out of 100 species-specific patterns were easily visualized in electrophoresis.

### Conversion of RAPD into SCAR and SNP

**Figure 2:**
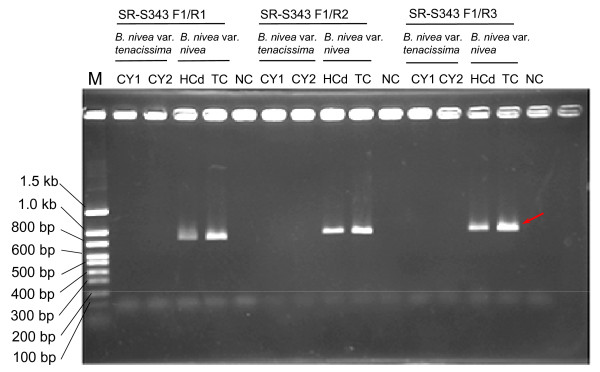
**SCAR band patterns of *B. nivea *var. *tenacissima *and *B. nivea *var. *nivea***. SCAR profiles of *Boehmeria *DNA fragments generated with primers SR-S343-F1, SR-S343-R1, SR-S343-R2 and SR-S343-R3. Representative samples of both species are shown on the top of lanes. The numbers on the left indicate the size of DNA standards. M: DNA markers; NC: negative control.

The SCAR reaction generated a unique band with *B. nivea *var. *nivea *DNA but no unique band with *B. nivea *var. *tenacissima *(Figure 2). SCAR analysis with primers developed from cloned variant-specific RAPD bands is highly specific. We identified three SCAR profiles with single bands easily visualized on agarose gels (Figure 2). As SCAR primers are sequence-specific, this method is less complex and more sensitive than RAPD. SCAR appears to be the method of choice for the characterization of mixtures of both *Boehmeria *variants in commercial herbal preparations.

**Figure 3:**
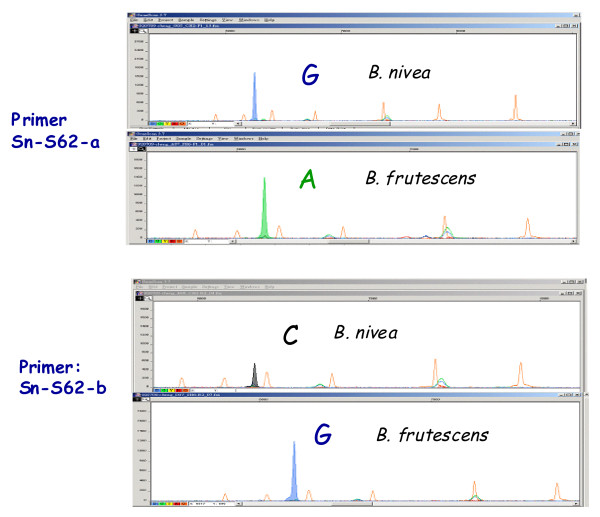
**Electropherogram of SNP markers for polymorphisms between *B. nivea *var. *tenacissima *and *B. nivea *var. *nivea***. The primer-extension reactions were performed by SNapshot with primers SNP-S62-a and SNP-S62-b, respectively. Polymorphisms between *B. nivea *var. *tenacissima *and *B. nivea *var. *nivea *are demonstrated by peaks with different colors (A = green, C = black and G = blue) at known locations. Orange peaks indicate positions of the LIS internal size standard (GeneScanTM-120).

The 0.6-kb RAPD fragment that generated from primer S62 was sequenced to identify species-specific SNPs. By using the SNaPshot as identification tools, the primers Sn-S62-f and Sn-S62-r indicated single nucleotide replacements of guanine to adenine and cytosine to guanine in *B. nivea *var. *nivea *and *B. nivea *var. *tenacissima*, respectively (Figure 3).

Only a fraction of the recommended SNaPshot mixtures was used in this study because the quality of SNP detection can be maintained using only one eighth of the recommended amounts of the reagents.

### Conversion of SNP sites into CAPS

**Figure 4:**
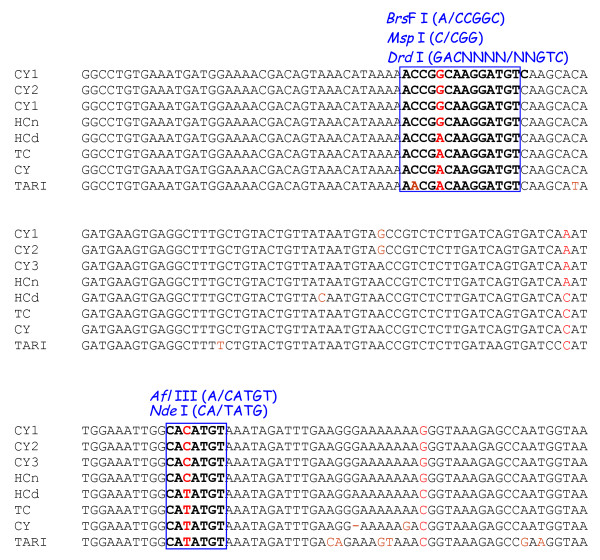
**Primer design for CAPS markers**. A 0.55-kb fragment was amplified from the eight *Boehmeria *lines with primers CP-S62-f (5'-CTGAGGCGGGAGCTAGGATTTCAACTAA-3') and CP-S62-r (5'-GGGGGAAGTAGTGCAGCACATGAATATA-3'). For the CAPS analysis, the restriction enzymes that generate polymorphic patterns were found by the dCAPS Finder 2.0 software (http://helix.wustl.edu/dcaps/dcaps.html). Five restriction enzymes (i.e. *Afl III*, *Bsr FI*, *Msp I*, *Drd I *and *Nde*) were used.

**Figure 5:**
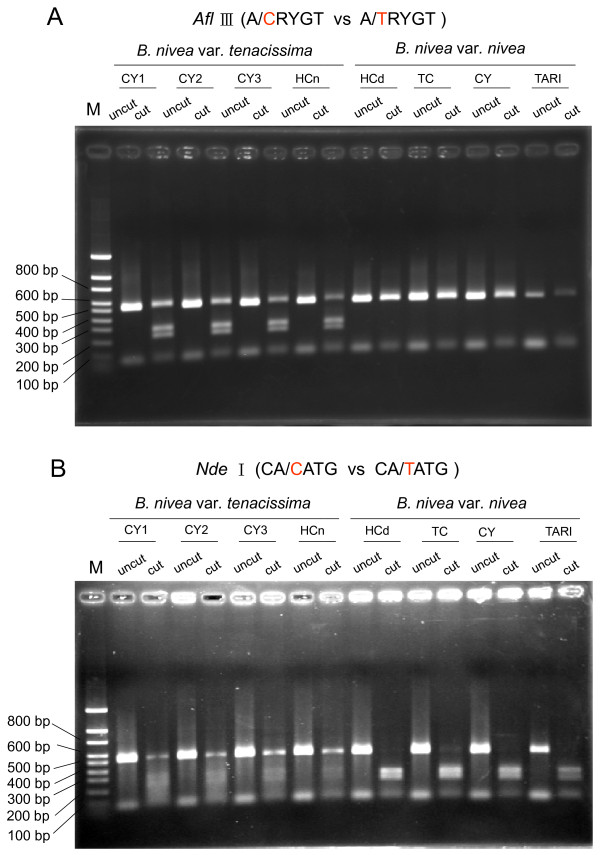
**Fragments of CAPs markers digested with *Afl *III and *Nde *I**. **A **CAPS profile generated by digestion with restriction enzyme *Afl *III. Representative samples of each *Boehmeria *lines are shown on top of the lanes. The numbers on the left indicate the size of DNA standards. M: DNA markers. **B **CAPS profile generated by digestion with restriction enzyme *Nde *I.

Sequence analysis of the S62-amplified fragments revealed several point mutations between the two *Boehmeria *species. Two of these mutation sites were selected for the CAPS assay, providing a total amount of five possible altered restriction enzyme sites (i.e. *Afl *III, *Bsr *FI, *Msp *I, *Drd *I and *Nde *I) between *B. nivea *var. *nivea *and *B. nivea *var. *tenacissima *(Figure 4). The modification of the sequencing protocol did not reduce the accuracy of the sequencing reaction required to identify species-specific SNPs. By digestion of the 0.55 kb RAPD fragments, i.e. Ca-Afl-0.55 and Ca-Nde-0.55 that amplified from the S62 primer and cut with *Afl *III and *Nde *I, we produced DNA fragments with predicted sizes of 0.30 kb and 0.25 kb for CAPS markers Ca-Afl-0.30 and Ca-Afl-0.25, and 0.35 kb and 0.20 kb for CAPS markers Ca-Drd-0.35 and Ca-Drd-0.20 that could be easily visualized. The species-specific patterns of CAPS markers digested with restriction enzyme *Afl *III and *Nde *I are shown in Figure 5. The CAPS markers clearly distinguished between the two *Boehmeria *species.

### Potential applications in quality control

**Figure 6:**
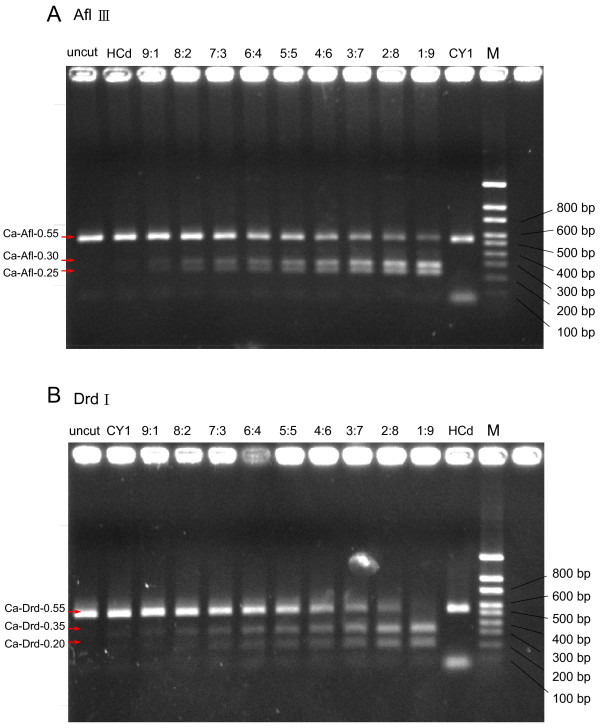
**Quality control of admixed DNA samples**. **A **Quality test of CAPS markers. Digestion pattern with restriction enzyme *Afl *III of the CAPS profile generated by admixed DNA samples from both *B. nivea *var. *tenacissima *(CY1) and *B. nivea *var. *nivea *(HCd) in the ratios of 9:1, 8:2, 7:3, 6:4, 5:5, 4:6, 3:7, 2:8 and 1:9. Representative samples of the DNA ratio between *B. nivea *var. *tenacissima *(CY1) and *B. nivea *var. *nivea *(HCd) were shown on top of the lanes. M: DNA markers. **B **Quality control of CAPS markers generated from restriction enzyme digestion with DNA samples from both *B. nivea *var. *nivea *(HCd) and *B. nivea *var. *tenacissima *(CY1) in the ratios of 9:1, 8:2, 7:3, 6:4, 5:5, 4:6, 3:7, 2:8 and 1:9. The digestion was performed with restriction enzyme *Drd *I.

To test the applicability of the markers, we selected CAPS analysis to trace the identification of components in preparations containing mixtures of DNA from both *Boehmeria *species. The results (Figure 6) indicated a shift in the electrophoretic patterns corresponding to the increasing and decreasing amounts of DNA from either species. The results confirmed the association of the Ca-Drd-0.55, Ca-Afl-0.30 and Ca-Afl-0.25 markers to *B. nivea *var. *nivea *and the Ca-Afl-0.55, Ca-Drd-0.35 and Ca-Drd-0.20 CAPS markers to *B. nivea *var. *tenacissima*.

Based on known SNPs, CAPS is a suitable strategy for the analysis of mixed samples. The present study identified the *Boehmeria *species in a sample and characterized the relative amounts of one species vs. another in a single sample. While CAPS may be used as a rapid analysis kit for *Boehmeria*-based preparation compared with the time-consuming RFLP analysis, however it is rather laborious and requires the use of restriction enzymes.

## Conclusion

The present study demonstrates the usefulness of the RAPD, SCAR, SNP and CAPS markers for rapid identification of variants between two closely related *Boehmeria *species. In particular, CAPS would be a suitable strategy for the analysis of mixed samples.

## Abbreviations

RAPD: random amplified polymorphic DNA; SCARs: sequence characterized amplified regions; SNP: single nucleotide polymorphism; CAPS: cleaved amplified polymorphic sequences; CY1: Chi-Yi-1; CY2: Chi-Yi-2; CY3: Chi-Yi-3; HCn: Hsin-Chu-n; HCd: Hsin-Chu-d; TC: Tai-Chung; CY: Chi-Yi; TARI: Taiwan Agricultural Research Institute; CTAB: hexadecyl trimethylammonium bromide; PVPP: polyvinylpolypyrrolidone; PCR: polymerase chain reaction

## Competing interests

The authors declare that they have no competing interests.

## Authors' contributions

CMC conceived and designed the study. CIL performed the laboratory work and data acquisition. SJC interpreted the data. TST collected and authenticated the plant samples. CYL drafted the manuscript. LTL finalized the manuscript. All authors read and approved the final version of the manuscript.
